# Study on the Application Value of Concurrent Chemoradiotherapy and Clinical Nursing Pathway for Postoperative Patients with Esophageal Cancer

**DOI:** 10.1155/2022/2216529

**Published:** 2022-09-15

**Authors:** Jinglei Yang, Xianzhong Zhang, Guangrong Yang, Xiufang Mi, Yang Zhang, Yingzhong Sui

**Affiliations:** ^1^Department of Radiophysics, The Affiliated Qingdao Central Hospital of Qingdao University, The Second Affiliated Hospital of Medical College of Qingdao University, Qingdao 266042, China; ^2^Department of Thoracic Surgery, The Affiliated Qingdao Central Hospital of Qingdao University, The Second Affiliated Hospital of Medical College of Qingdao University, Qingdao 266042, China; ^3^Department of Oncology (I), The Affiliated Qingdao Central Hospital of Qingdao University, The Second Affiliated Hospital of Medical College of Qingdao University, Qingdao 266042, China; ^4^Community Health Service Center of Government District, Zhangqiu District People's Hospital, Jinan 250200, China; ^5^Department of Respiratory, Zhangqiu District People's Hospital, Jinan 250200, China

## Abstract

**Backgrounds:**

To observe the value of concurrent chemoradiotherapy and clinical nursing pathway for postoperative patients with esophageal cancer (EC).

**Methods:**

A total of 88 postoperative EC patients were divided into the radiotherapy group (RG group, 44 cases) and the chemoradiotherapy group (CRG group, 44 cases). The RG group received single three-dimensional conformal radiotherapy+clinical nursing pathway, and the CRG group was combined with chemotherapy on this basis. The 5-year overall survival rate, progression-free survival rate, pathological remission and survival rate, lymph node metastasis and survival rate, quality of life analysis, tumor-related factor level, and incidence of adverse reactions were compared between the two groups.

**Results:**

The overall survival rates at 1, 3, and 5 years were 93.18%, 56.82%, and 50.0% in the CRG group and 86.36%, 52.27%, and 43.18% in the RG group, respectively. The 5-year progression-free survival rate of the CRG group was 60.87%, which was clearly higher than that of the RG group (33.33%). The 1-, 3-, and 5-year overall survival rates of pCR and NpCR patients were 90.48%, 80.95%, and 61.90% and 89.55%, 44.78%, and 38.81%, respectively. The overall 1-year, 3-year, and 5-year survival rates were 81.08%, 37.84, and 24.32% and 96.08%, 66.67%, and 62.75% in patients with lymph node metastasis and nonlymph node metastasis, respectively, with statistical significant differences. The emotional function, physical function, cough, pain, and eating difficulty in the CRG group were better than those in the RG group. After treatment, serum CEA, SCC, CYFRA21-1, and CA199 levels in the CRG group were obviously downregulated compared with those in the RG group. There was no obvious difference in the incidence of adverse reactions between the CRG group and the RG group.

**Conclusion:**

Single radiotherapy and concurrent chemoradiotherapy can be used as effective means in the treatment of EC. Moreover, the quality of life and survival time of the concurrent chemoradiotherapy group were dramatically better than those of the single radiotherapy group, and the antitumor ability of the concurrent chemoradiotherapy group was stronger.

## 1. Introduction

Esophageal cancer (EC) is a common malignant tumor, causing about 300,000 deaths worldwide every year [1]. China is one of the regions with high incidence of EC in the world, and about 150,000 people die of esophageal cancer every year [2]. The typical symptoms of EC are progressive hypopharyngeal difficulty, retrosternal pain, and finally cachexia [3]. Clinically, it is believed that the incidence of EC is related to environmental and dietary habits and other factors [4]. Surgical treatment is preferred for patients with good basic condition and no obvious metastasis. However, the 5-year survival rate of locally advanced EC with surgery alone and radiotherapy alone is about 20%-30% [5]. The main reasons for the failure are local recurrence and metastasis, and the recurrence sites are mainly in anastomotic sites and lymph nodes. The second operation is difficult, traumatic, and the cure rate is low, and most patients cannot tolerate it. Therefore, the comprehensive treatment based on radiotherapy and chemotherapy is often adopted to remove residual tumor cells and prolong the life of patients. Practice has proved [6–8] that although concurrent radiotherapy and chemotherapy can prolong the lives of most patients with esophageal cancer, due to its strong cytotoxicity, it will not only reduce the immune function of patients but also bring strong adverse reactions to patients, seriously affecting the lives of patients, and some patients give up treatment due to unbearable adverse reactions. In order to make esophageal cancer patients recover smoothly after operation and reduce the occurrence of adverse reactions, it is urgent to adopt a set of effective clinical nursing methods.

In this study, clinical nursing pathway [9] was applied to postoperative radiotherapy and chemotherapy nursing of esophageal cancer, which is of great significance for postoperative rehabilitation.

## 2. Materials and Methods

### 2.1. General Information

A total of 88 patients with EC treated in our hospital from March 2015 to November 2016 after radical resection were retrospectively analyzed. All patients were diagnosed as esophageal squamous cell carcinoma by surgical pathology, without tumor residue after radical resection. There were 57 males and 31 females, aged 42-78 years, with a median age of 62 years. According to the different treatment schemes, they were divided into two groups, with 44 cases in each group. The chemoradiotherapy (CRG) group received 3DCRT combined with chemotherapy regimen of TS-1 combined with oxaliplatin+clinical care pathway. The single radiotherapy (RG) group received 3DCRT+clinical care pathway. There were no statistically significant differences in gender, age, KPS score, and other clinical data between two groups (all *P* > 0.05, Table 1).

Inclusion criteria: (1) all were confirmed as EC by pathology or imaging; (2) no previous chest radiotherapy, no contraindication of radiotherapy and chemotherapy; (3) KPS score ≥ 70, estimated survival time ≥ 6 months, with normal blood routine, liver, and kidney function and electrocardiogram; and (4) the study was approved by our hospital's ethics committee, and the patients signed the informed consent for treatment

Exclusion criteria: (1) associated with other cancers or tumors, (2) with autoimmune diseases, and (3) poor compliance.

### 2.2. Therapeutic Method

#### 2.2.1. 3DCRT

The patients were fixed in the thermoplastic film position and positioned by enhanced CT scanning. The images were transmitted to the treatment planning system (TPS) to delineate the tumor target area, that is, the gross tumor volume (GTV). GTV referred to enlarged lymph nodes (lymph node short diameter ≥ 10 mm). On the basis of GTV, the clinical target volume (CTV) was expanded by 5-8 mm from front to back, left to right, and 8-10 mm from top to bottom. Based on CTV, planning target volume (PTV) was expanded uniformly by 5 mm. The prescribed dose was 60-64 Gy, the median dose was 62 Gy, and the fractional dose was 1.8~2.0 Gy/time, 5 times/week.

#### 2.2.2. Chemotherapy

Chemotherapy was performed on a regimen of TS-1 combined with oxaliplatin. 40 mg/m^2^ tegafur/gimeracil/oteracil was taken orally once in the morning and once in the evening, *d*_1–14_. 130 mg/m^2^ of oxaliplatin was given intravenously, *d*_1_. One cycle was 21 days. The CRG group received 2 cycles of chemotherapy during radiotherapy. After radiotherapy, the two groups were given chemotherapy of the above scheme for 2~4 cycles according to their condition. In the course of chemotherapy, antiemetic, acid inhibition, liver protection, and symptomatic support were given. Cold stimulation should be avoided during the application of oxaliplatin to reduce the occurrence of peripheral neurotoxicity.

### 2.3. Clinical Nursing Pathway [10]

#### 2.3.1. The First Stage

A clinical nursing pathway group was set up and led by the department head and head nurse. Relevant nurses were trained to fully understand the importance of clinical nursing pathways.

#### 2.3.2. The Second Stage

The team members take the patients as the nursing center, followed the principles of scientificity, practicality, and comprehensiveness, and formulated a clinical nursing path plan including doctors, nurses, and patients. The details are as follows:

1. Oral care was taken twice a day to avoid oral erosion. The nursing staff wiped the patient's body with warm water once a day to prevent pressure sores and timely treated the nausea and vomiting caused by radiotherapy and chemotherapy. Conscious patients were guided to perform appropriate active and passive exercises on the upper and lower limbs of the operation side. Besides, abdominal breathing exercises were carried out to enhance their respiratory ability, so as to effectively improve their respiratory ability and alleviate their limb stiffness
2. The nursing staff should monitor the patient's vital signs and drainage tube and replace the drainage tube in time. Patients were encouraged to urinate on their own, actively carry out daily activities such as limb movement, dressing, and bathing, and instructed to correct lying posture and drink a small amount of water. Intuitive videos and manuals were made, lectures were held regularly, and “one-to-one” explanations were conducted to improve patients' awareness of the occurrence and development, influencing factors, precautions, and treatment measures, to enhance patients' treatment confidence and improve treatment compliance
3. After no abnormality in drinking a small amount of water, the patients can be instructed to eat rice soup, chicken soup, and other liquid foods. Patients were instructed to eat semiliquid food such as porridge to ensure their normal nutrition and avoid spicy food, tobacco, and alcohol. Patients were instructed to take appropriate outdoor exercise to maintain a pleasant mood

#### 2.3.3. The Third Stage

As the clinical nursing pathway is a new nursing quality management mode, which conforms to the relevant laws of management, it is necessary to timely understand the latest application and progress at home and abroad. In addition, the clinical nursing pathway scheme formulated by our hospital was modified and improved, so as to effectively improve the overall nursing quality and provide good nursing services for patients to the greatest extent.

### 2.4. Observation Index

Adverse reactions were based on RTOG standard [11] and CTCAE 4.0 standard [12], including radioactive esophagitis, radioactive pneumonia, and peripheral neurotoxic reactions.

Patients were followed up 6 months after the end of treatment and evaluated by the quality of life after esophageal cancer scale [13] (QLQ-OES18) and the quality of life scale developed by the European Organization for Research and Treatment of Cancer (QLQ-C30) [14].

Overall survival (OS) was defined as the time from enrollment to death from any cause or the last follow-up. The patients in the two groups were reexamined by chest CT, esophageal barium swallowing radiographs, and abdominal ultrasound 1-3 months after the end of radiotherapy in both groups, once every 3 months, and once every 6 months in the second year. The starting point of follow-up observation was from the day of admission, and the follow-up was carried out by outpatient review and telephone. The survival of patients at 1, 3, and 5 years during the follow-up period was observed and recorded.

Seven days after treatment, the levels of CEA, SCC, CYFRA21-1, and CA199 were detected by ELISA. The detection was carried out in accordance with the requirements of the kit instructions.

### 2.5. Statistical Analysis

SPSS 19 0 statistical software was used for data processing. The counting data were expressed as *n* (%) and analyzed by using the *χ*^2^ test. The measurement data were expressed as mean ± standard deviation and analyzed by *t*-test. The survival rate was calculated by Kaplan-Meier method and log-rank test. *P* < 0.05 was considered as significant difference.

## 3. Results

### 3.1. Comparison of Overall Survival Rate and Progression-Free Survival Rate

**Figure 1:**
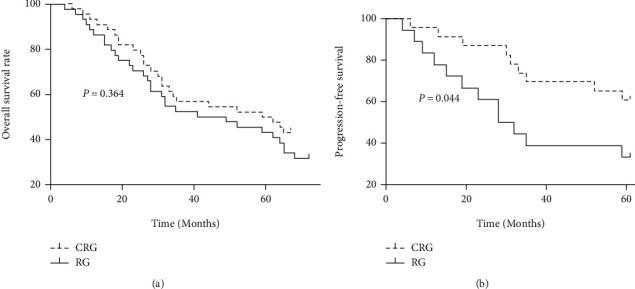
Overall survival and progression-free survival curves. (a) Overall survival curve. (b) Progression-free survival curve.

The CRG group was followed up for 6-67 months, with a median follow-up of 47 months. The RG group was followed up for 4-72 months, with a median follow-up of 44 months. The overall survival rates at 1, 3, and 5 years were 93.18% (41/44), 56.82% (25/44), and 50.0% (22/44) in the CRG group and 86.36% (38/44), 52.27% (23/44), and 43.18% (19/44) in the RG group, respectively. Postoperative chemotherapy did not significantly improve overall survival after neoadjuvant chemoradiotherapy (Figure 1(a)). At the end of follow-up, 21 patients in the CRG group and 26 patients in the RG group were at the disease progression stage. The 5-year progression-free survival rate in the CRG group was 60.87%, significantly higher than that in the RG group (33.33%). Postoperative chemotherapy improved progression-free survival in patients who have received neoadjuvant chemoradiotherapy combined with surgery (Figure 1(b)).

### 3.2. Survival Curve Analysis of Pathological Remission

**Figure 2:**
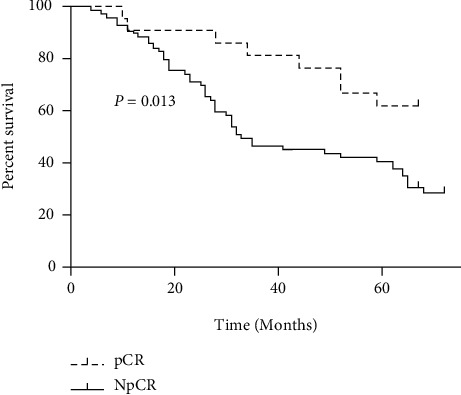
The survival curve of the pCR group and the NpCR group.

In all 88 patients, pCR rate was 23.86% (21/88). The 1-, 3-, and 5-year overall survival rates of pCR and NpCR patients were 90.48% (19/21), 80.95% (17/21), and 61.90% (13/21) and 89.55% (60/67), 44.78% (30/67), and 38.81% (26/67), respectively. The difference was statistically significant (Figure 2).

### 3.3. Survival Curve Analysis of Lymph Node Metastasis

**Figure 3:**
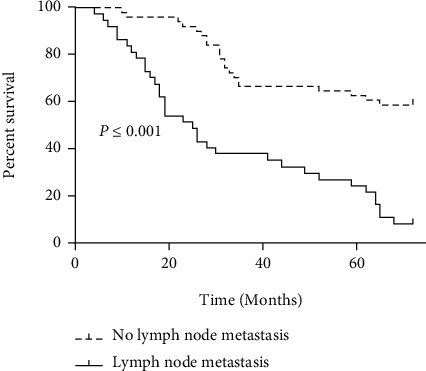
Survival curves of the lymph node metastasis group and the nonlymph node metastasis group.

In all 88 patients, the lymph node metastasis rate was 42.05% (37/88). The 1-, 3-, and 5-year overall survival rates of patients with and without lymph node metastasis were 81.08% (30/37), 37.84 (14/37), and 24.32% (9/37) and 96.08% (49/51), 66.67% (34/51), and 62.75% (32/51), respectively (Figure 3).

### 3.4. Comparison of Life Quality

In the QLQ-C30 scale, emotional function and physical function of patients in the CRG group were better than those in the RG group (both *P* < 0.05). In the QLQ-OES18 scale, cough, eating pain, and eating difficulty in the CRG group were better than those in the RG group (all *P* < 0.05). There was no statistical difference in other evaluation indexes (Tables 2 and 3).

### 3.5. Comparison of Levels of Tumor-Related Factors

**Figure 4:**
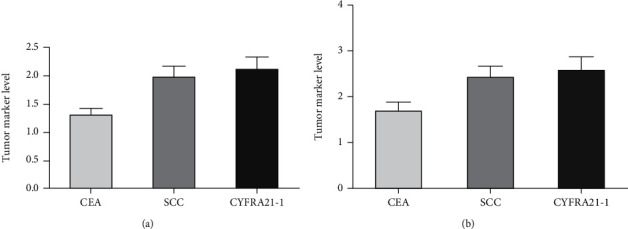
CEA, SCC, and CYFRA21-1 levels were compared between the two groups after treatment. (a) CEA, SCC, and CYFRA21-1 levels in the CRG group after treatment. (b) CEA, SCC, and CYFRA21-1 levels in the RG group after treatment.

**Figure 5:**
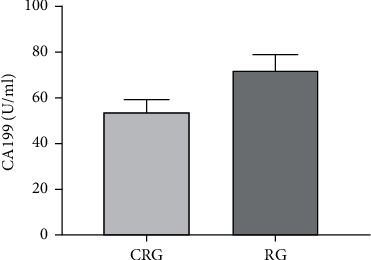
Comparison of CA199 levels between the two groups after treatment.

After treatment, serum CEA, SCC, CyFRA21-1, and CA199 levels in the CRG group were clearly downregulated compared with those in the RG group, and the differences were statistically significant (all *P* < 0.05, Figures 4 and 5).

### 3.6. Comparison of the Occurrence of Adverse Reactions

**Figure 6:**
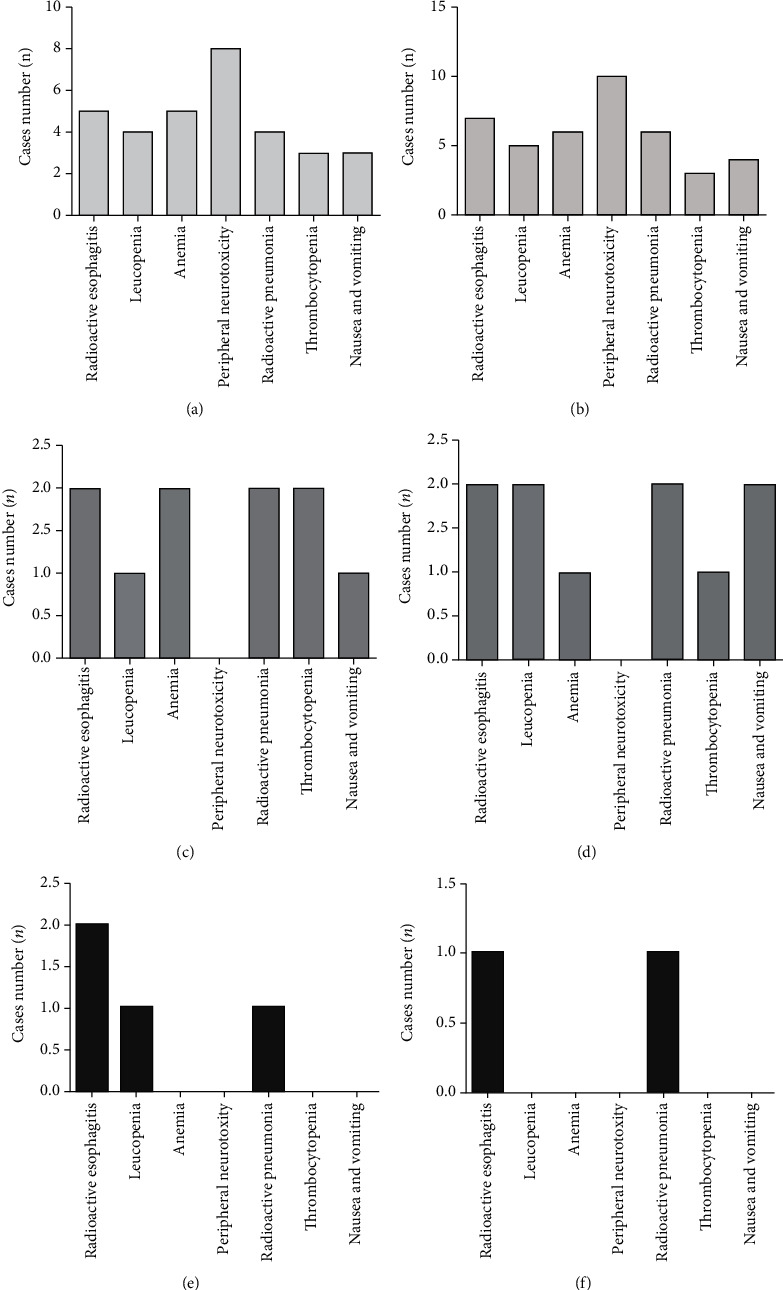
The incidence of adverse reactions was compared between the two groups. (a) Occurrence of various adverse reactions in the CRG group (I). (b) Occurrence of various adverse reactions in the RG group (I). (c) Occurrence of various adverse reactions in the CRG group (II). (d) Occurrence of various adverse reactions in the RG group (II). (e) Occurrence of various adverse reactions in the CRG group (III). (f) Occurrence of various adverse reactions in the RG group (III).

Patients in both groups had nausea and vomiting, radioactive esophagitis, radioactive pneumonia, peripheral neurotoxicity, leukopenia, anemia, and thrombocytopenia. Patients were well tolerated after active symptomatic support treatment during radiotherapy and chemotherapy. There was no obvious difference in the incidence of adverse reactions between the CRG group and the RG group (all *P* > 0.05, Figure 6).

## 4. Discussion

The incidence of EC ranks the 6th among malignant tumors, and the fatality rate ranks the 4th, with an obvious upward trend in recent years [15]. For patients with resectable EC, surgery is still the most important treatment. However, the effect of single treatment was poor, with a high postoperative local recurrence rate, and the 5-year postoperative survival rate was lower than 25% [16]. After surgery, concurrent radiochemotherapy were required [17] to completely remove residual lesions or tumor cells. As a new oral fluorouracil drug, tegafur/gimeracil/oteracil has excellent oral bioavailability with a half-life of 12 hours, which can enhance the antitumor effect and reduce gastrointestinal adverse reactions [18]. At present, tegafur has been widely used in Japan for the treatment of advanced gastric cancer, colorectal cancer, and head and neck tumors, including EC [19]. Guo et al. [20] reported a clinical study on the treatment of locally advanced EC with tegafur/gimeracil/oteracil+cisplatin chemotherapy combined with radiotherapy and achieved good efficacy and pathological complete response rate. Oxaliplatin, as a third-generation platinum-based chemotherapy agent, has stronger DNA inhibition and cytotoxicity than DDP and no cross-resistance with DDP and carboplatin [21]. Oxaliplatin has high anticancer activity, mild nephrotoxicity and bone marrow inhibition, and with only some neurotoxicity. FOLFOX4 has been applied to synchronous radiotherapy for EC, with definite efficacy and relatively mild adverse reactions [22].

Effective nursing intervention is an important means to improve the quality of life of EC patients [23]. Clinical nursing pathway has been widely used in brain department, liver department, and critical ICU and achieved better nursing effects [24]. Clinical nursing path refers to the treatment and nursing mode developed by specialists, responsible nurses, psychological consultants, and dietitians according to diseases or surgeries and approved by relevant medical staff. It is synthesized from the routine nursing plan of each diagnosis, which can guide the nurses to work proactively and predictably. At the same time, it also enables the patients to clarify their own nursing goals and consciously participate in the nursing process of the disease [25].

In this study, QLQ-C30 and QLQ-OES18 scales were used to comprehensively investigate the quality of life of patients 6 months after the end of treatment. There was no statistical difference in the scores of speech function, dry mouth, anorexia, and other aspects between the two groups, and the CRG group had the advantage of fast recovery after the end of treatment. Patients in the two groups had similar dysphagia and obstruction, indicating that synchronous chemoradiotherapy could also solve the problem of eating obstruction compared with postoperative radiotherapy. All indexes of the two groups were notably improved compared with before treatment, indicating that postoperative single radiotherapy and concurrent chemoradiotherapy combined with clinical nursing path have better therapeutic effect on patients with EC and can improve the quality of life.

There was no significant difference in overall survival at 1, 3, and 5 years between the two groups, but the 5-year progression-free survival rate in the CRG group was obviously higher than that in the RG group. Although postoperative chemotherapy can significantly reduce the lesions, relieve symptoms, and prolong survival, it did not notably improve the overall survival rate, and the overall remission rate was not high suggesting that the CRG group may obtain similar efficacy as the RG group.

Tumor-related factors are important indicators to evaluate the curative effect of clinical treatment for malignant tumors. As a glycoprotein, CEA is mostly found on the surface of tumor cells and can participate in cell deterioration, proliferation, and metastasis by damaging epithelial tissues and improving the adhesion ability of tumor cells [26]. SCC is a specific antigen generated during the proliferation of squamous cell carcinoma, which has high specificity and sensitivity to squamous cell carcinoma and can be used for the diagnosis and prognosis evaluation of squamous cell carcinoma [27]. CYFRA21-1 is an important component of tumor cytoskeleton and widely exists in the cytoplasm. When EC cells proliferate and expand abnormally, CYFRA21-1 can be synthesized in large quantities and released into the blood by exocrine [28]. CA199 [29] is involved in the formation of tumor cells and can be released into the blood when tumor cells die, rupture, or proliferate, resulting in abnormal elevation of their serum level. Our results indicated that the levels of CEA, SCC, CYFRA21-1, and CA199 in the CRG group were lower than those in the RG group after treatment, indicating that synchronous chemoradiotherapy can promote the apoptosis of malignant tumor cells and suppress the generation of tumor-related factors compared with single chemoradiotherapy.

## 5. Conclusion

Above all, the application of concurrent chemoradiotherapy combined with clinical nursing pathway in postoperative treatment of EC patients can promote the apoptosis of EC cells, inhibit the generation of tumor-related factors, and improve the quality of life and the efficacy.

## Figures and Tables

**Table 1:** Comparison of general data between the two groups.

	*n*	CRG (44)	RG (44)	*χ* ^2^/*t*	*P*
Gender				0.050	0.823
Male	57	28	29		
Female	31	16	15		
Age (years)				1.203	0.232
Age range	42-78	42-74	44-78		
Median age	62	61	65		
Average age	61.59 ± 9.06	60.43 ± 8.08	62.75 ± 9.90		
KPS score				1.245	0.536
70	22	12	10		
80	35	19	16		
90	31	13	18		
Location				0.561	0.756
Upper thoracic	5	3	2		
Midthoracic	65	31	34		
Lower thoracic	18	10	8		
T stage				0.563	0.453
T_3_	21	9	12		
T_4_	67	35	32		
N stage				0.095	0.368
N_0_	30	13	17		
N_1_	58	31	27		
Tumor differentiation				1.693	0.429
High	43	19	24		
Moderate	36	19	17		
Low	9	6	3		

**Table 2:** Comparison of QLQ-OES18 score between two groups.

		Speech function	Cough	Appetite decreases	Xerostomia	Obstruction
Before treatment	CRG (44)	25.86 ± 7.81	33.36 ± 8.76	37.23 ± 9.51	32.32 ± 7.48	34.09 ± 8.11
	RG (44)	28.05 ± 9.48	34.48 ± 7.44	34.14 ± 8.16	33.43 ± 6.27	31.07 ± 8.57
*t*		1.178	0.643	1.636	0.757	1.699
*P*		0.242	0.522	0.106	0.451	0.093
After treatment	CRG (44)	20.09 ± 8.32	21.25 ± 9.62	25.11 ± 9.15	20.93 ± 7.00	23.02 ± 7.39
	RG (44)	21.09 ± 7.90	31.14 ± 8.70	23.73 ± 8.95	21.23 ± 6.21	25.30 ± 7.24
*t*		0.578	5.054	0.760	0.210	1.458
*P*		0.565	0.001	0.450	0.835	0.149

		Swallow saliva	Eating pain	Reflux	Eating difficulty	Dysphagia

Before treatment	CRG (44)	30.89 ± 6.59	36.02 ± 7.50	35.09 ± 8.11	37.23 ± 9.51	31.95 ± 6.99
	RG (44)	31.18 ± 6.78	35.18 ± 6.78	32.91 ± 7.52	36.14 ± 8.15	31.00 ± 7.33
*t*		0.207	0.482	1.309	0.578	0.625
*P*		0.836	0.631	0.194	0.565	0.534
After treatment	CRG (44)	16.82 ± 6.25	12.82 ± 6.27	20.16 ± 8.78	25.07 ± 7.98	21.82 ± 6.42
	RG (44)	16.91 ± 4.74	16.23 ± 6.09	23.09 ± 7.88	19.86 ± 6.44	21.14 ± 8.70
*t*		0.066	2.587	1.648	3.366	1.422
*P*		0.948	0.011	0.103	0.001	0.159

**Table 3:** Comparison of QLQ-C30 score between two groups.

		Social function	Cognitive function	Emotional function	Role function	Physical function
Before treatment	CRG (44)	63.09 ± 7.98	64.73 ± 9.05	60.91 ± 7.05	63.80 ± 7.17	63.16 ± 8.52
	RG (44)	64.66 ± 8.33	67.09 ± 8.05	62.02 ± 7.25	61.89 ± 6.51	61.09 ± 8.14
*t*		0.902	1.294	0.731	1.308	1.164
*P*		0.370	0.199	0.467	0.194	0.248
After treatment	CRG (44)	79.93 ± 12.34	80.75 ± 10.31	75.16 ± 8.78	76.64 ± 11.11	81.73 ± 9.18
	RG (44)	82.64 ± 10.29	77.23 ± 9.51	67.09 ± 8.11	79.00 ± 12.41	73.50 ± 11.10
*t*		1.117	1.666	4.478	0.941	3.789
*P*		0.267	0.099	≤0.001	0.349	≤0.001

## Data Availability

The data used to support the findings of this study are available from the corresponding author upon request.
